# Glutamate induces autophagy via the two-pore channels in neural cells

**DOI:** 10.18632/oncotarget.14404

**Published:** 2016-12-31

**Authors:** Gustavo J.S. Pereira, Manuela Antonioli, Hanako Hirata, Rodrigo P. Ureshino, Aline R. Nascimento, Claudia Bincoletto, Tiziana Vescovo, Mauro Piacentini, Gian Maria Fimia, Soraya S. Smaili

**Affiliations:** ^1^ Department of Pharmacology, Federal University of São Paulo, (UNIFESP), São Paulo, Brazil; ^2^ Department of Biology, University of Rome “Tor Vergata”, Rome, Italy; ^3^ Department of Epidemiology and Preclinical Research, National Institute for Infectious Diseases IRCCS 'Lazzaro Spallanzani', Rome, Italy; ^4^ Department of Biological and Environmental Sciences and Technologies (DiSTeBA), University of Salento, Lecce, Italy

**Keywords:** autophagy, glutamate, NAADP, two-pore channels, AMPK

## Abstract

NAADP (nicotinic acid adenine dinucleotide phosphate) has been proposed as a second messenger for glutamate in neuronal and glial cells via the activation of the lysosomal Ca^2+^ channels TPC1 and TPC2. However, the activities of glutamate that are mediated by NAADP remain unclear. In this study, we evaluated the effect of glutamate on autophagy in astrocytes at physiological, non-toxic concentration. We found that glutamate induces autophagy at similar extent as NAADP. By contrast, the NAADP antagonist NED-19 or SiRNA-mediated inhibition of TPC1/2 decreases autophagy induced by glutamate, confirming a role for NAADP in this pathway. The involvement of TPC1/2 in glutamate-induced autophagy was also confirmed in SHSY5Y neuroblastoma cells. Finally, we show that glutamate leads to a NAADP-dependent activation of AMPK, which is required for autophagy induction, while mTOR activity is not affected by this treatment. Taken together, our results indicate that glutamate stimulates autophagy via NAADP/TPC/AMPK axis, providing new insights of how Ca^2+^ signalling glutamate-mediated can control the cell metabolism in the central nervous system.

## INTRODUCTION

Macroautophagy (here after referred as autophagy) is the process by which damaged or superfluous intracellular constituents are engulfed in double-membrane vesicles, named autophagosomes, and delivered to the lysosomes for degradation and recycling [1, 2].

Autophagosomes arise from phosphatidylinositol-3-phosphate-rich microdomains (omegasomes) generated by the Beclin 1/VPS34 complex, mainly located on the endoplasmic reticulum [3]. LC3 (microtubule associated protein 1A/1B light chain 3) family proteins label autophagosomes, playing an essential role both in vesicle elongation/closure and cargo recognition. In low autophagy conditions, LC3 is diffused in the cytosol (LC3-I isoform), while, following autophagy induction, LC3 is covalently modified by the addition of a phosphatidylethanolamine (LC3-II isoform), which allows its translocation to the autophagosome membrane where it remains associated until fusion to lysosomes [4, 5].

Acidification is an important step in the activation of lysosomal enzymes, which allows to generate functional autolysosomes and to promote a complete degradation of autophagic substrates [5, 6]. The lysosomal pH is maintained at around pH 4.5 by proton pumps that transport H^+^ ions into lysosomes [4]. The inhibition of lysosomal activity results in the accumulation of “undigested” lipidated LC3-II. To discriminate between increased autophagosome formation and reduced lysosomal activity, LC3II has to be analysed by comparing the levels in the untreated cells with respect to the one treated with lysosomal inhibitors (*e.g*. E64d and Pepstain A) to measure the rate of autophagosome degradation also defined as autophagic flux [4].

Ca^2+^ signalling have a crucial role in many aspects of lysosomal function [7–9]. An increase of cytosolic Ca^2+^ is necessary for the fusion of lysosomes to different types of vesicles, including phagosomes, late endosomes, and autophagosomes [10–16], as well as with the plasma membrane [17]. Furthermore, membrane trafficking during endocytosis and autophagy involves many fusion and fission events that are regulated by Ca^2+^ [11, 18].

Interestingly, lysosomes are also Ca^2+^ storage organelles, and their relevance in intracellular signalling is now gaining attention [19]. Indeed, the Ca^2+^ concentration in lysosomes is high and is partially dependent on the H^+^ gradient [17]. Recently, was identified a vertebrate Ca^2+^/H^+^ exchanger (CAX), located at acidic compartments, widespread expressed, evoking Ca^2+^signals, and regulating cell-matrix adhesion during cell migration [20]. Importantly, a family of Ca^2+^ channels, known as the two-pore channels (TPCs), have emerged as potential regulators of Ca^2+^ homeostasis [21]. The TPCs localise at endosomes and/or lysosomes through an identified targeting motif and regulate NAADP-mediated cytosolic Ca^2+^ signals [11, 21–23]. In fact, the inhibition of TPC expression/function using siRNA [23], knockout mice [24], and dominant-negative TPC constructs [22], reduces NAADP-evoked Ca^2+^ signals. Recently, a role for NAADP/TPC signalling in the regulation of autophagy has been identified. NAADP can induce autophagy in astrocytes [11], whereas treatments disturbing TPC-mediated Ca^2+^ release in mouse embryonic fibroblasts lead to a reduction of autophagy [25]. By contrast, TPC2 overexpression may cause a block of autophagosome-lysosome fusion in HeLa cells by preventing the recruitment of Rab-7 to autophagosomes [26].

Interestingly, a recent report described the agonist-specific recruitment of NAADP-sensitive Ca^2+^stores by glutamate; however, the molecular mechanisms underlying this event are still not known [27]. To gain insights on this signalling pathway, we investigated the effect of glutamate on autophagy and its crosstalk with the NAADP/Ca^2+^ signalling pathway, focusing on the role of the lysosomal Ca^2+^-permeable two-pore channels (TPCs).

## RESULTS

### Glutamate induces autophagic flux via NAADP

We have previously shown that NAADP treatment induces autophagy by mobilizing Ca^2+^ from acidic Ca^2+^ stores through TPCs. Since NAADP has been proposed as a second messenger for glutamate [27], we asked if glutamate might modulate autophagy via NAADP signalling.

**Figure 1:**
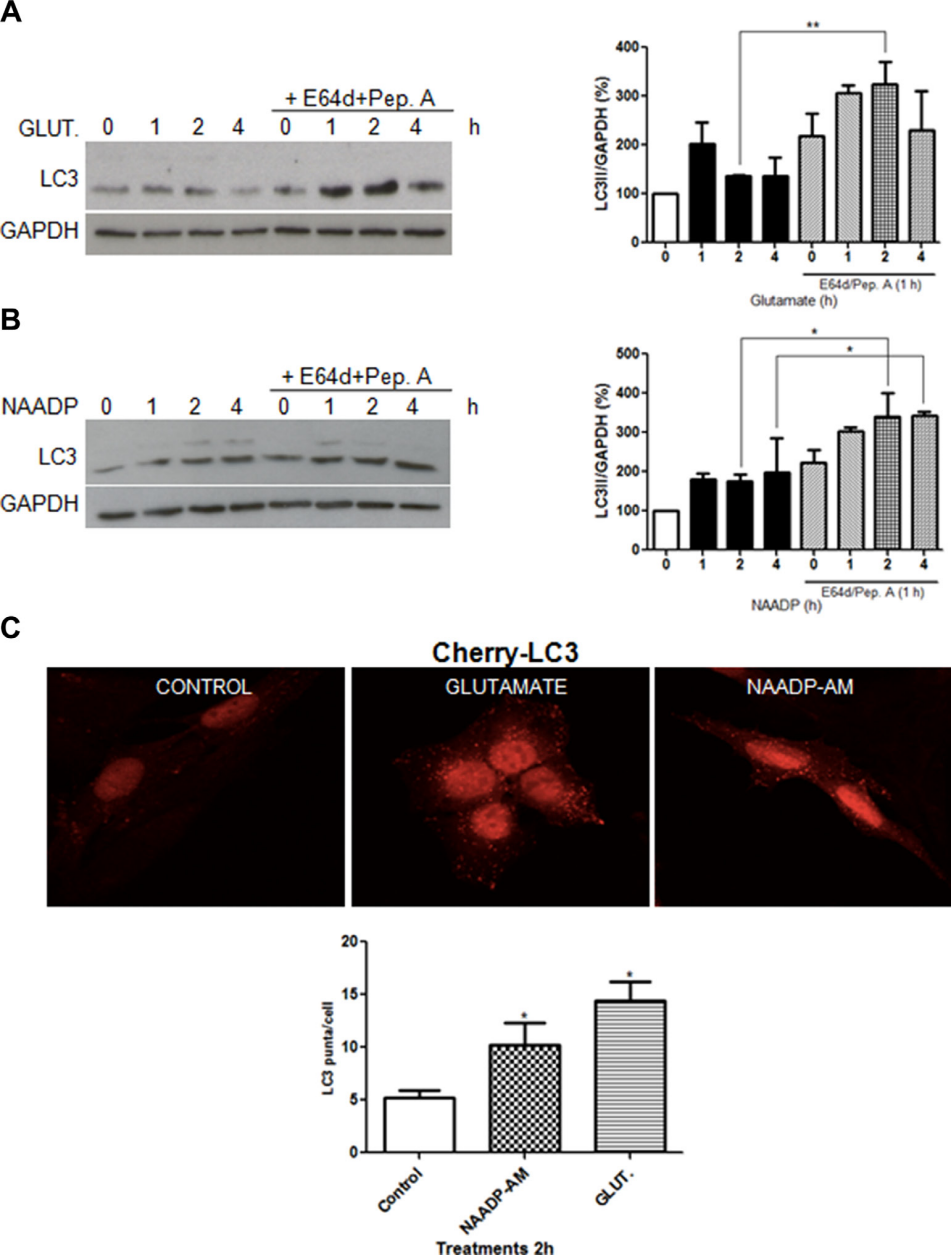
Glutamate and NAADP induce autophagy in astrocytes LC3 was detected by western blotting in cells treated with glutamate (10 μM) **(A)**, NAADP-AM (100 nM) **(B)** in the presence or absence of the lysosomal inhibitors E64d/pepstatin A (10 μg/mL, 1 h) at the indicated time for 1, 2, and 4 h for each treatment. Samples were subjected to western blotting using anti-LC3 and anti-GAPDH antibodies. Representative images of LC3-II are shown (left panel). Graph (right panel) reports means ± s.e.m. of LC3-II levels after GAPDH normalization from three independent experiments; *p < 0.05, **p < 0.01. LC3-II levels in control cells were set as 100. **(C)** Autophagosomes numbers were analysed in astrocytes expressing Cherry-LC3 incubated with NAADP-AM (100 nM) or glutamate (10 μM) for 2 h, by counting the number of mCherry puncta per cell (graph, lower panel). Representative fluorescent images are shown (upper panels, scale bars, 20 μm). At least 40 cells were included for each group. *p < 0.05 in relation to ctrl group (one way ANOVA, followed by Dunnett post-test).

Modulation of autophagic flux by glutamate was analysed in immortalized rat astrocytes and SHSY5Y neuroblastoma cells, in comparison with NAADP. Cells were treated with 10 μM glutamate or 100 nM NAADP-AM, in the presence or absence of the lysosome inhibitors E64d/pepstatin A, and analysed by western blotting using an anti-LC3 antibody. Similar to NAADP, glutamate induced autophagy flux, as demonstrated by the increase of LC3-II signals in the presence of lysosomal inhibitors in astrocytes (Figure 1A–1B).

To provide additional evidence for the ability of glutamate to induce autophagy in astrocytes, we used a stable cell line expressing a fluorescent-tagged mCherry-LC3 [28]. Treatment with glutamate (10 μM) or NAADP-AM (100 nM) for 2 h resulted in an increase of red fluorescent puncta, confirming the formation of LC3 positive vesicles (Figure 1C).

**Figure 2:**
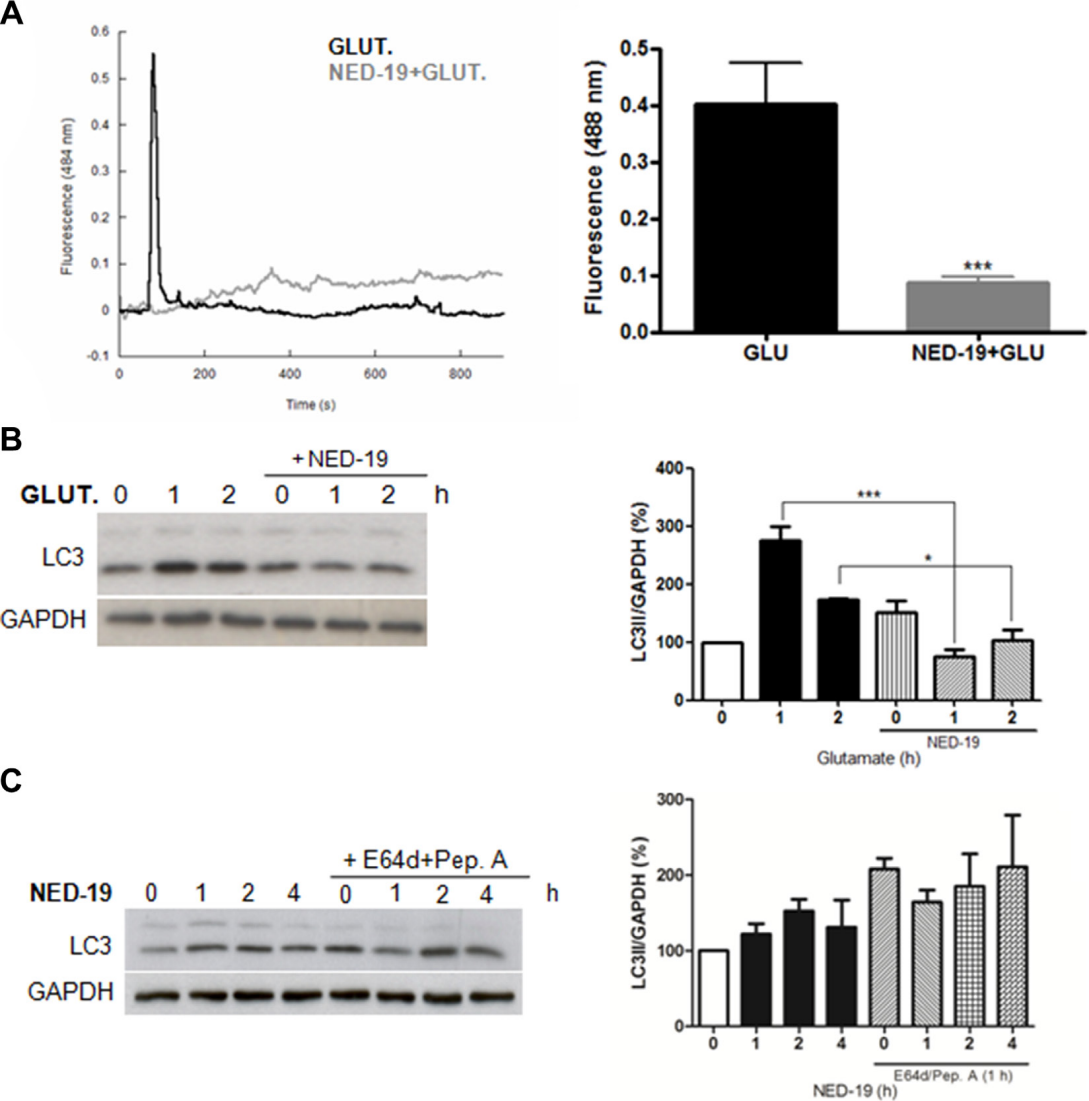
NED-19 inhibits Ca2+ release and autophagy induction by glutamate **(A)** Cytosolic Ca2+ responses of individual Fluo-4-loaded cells stimulated with 10 μM with glutamate (10 μM) with our without preincubation with NED-19 (1 μM, 30 min). Representative data are shown on the left panel (dark line: Glutamate treated cells in absence of NED-19; grey line: glutamate treated cells in presence of NED-19). Right panel: summary data quantifying the amplitude of the Ca2+ signals in the indicated cells. Data are expressed as mean ± s.e.m. All data are from at least 3 different experiments. ***p < 0.001. **(B)** LC3 level was detected by western blotting in astrocytes treated with glutamate (10 μM) in the presence or absence of NED-19 (1 μM). Samples were subjected to western blotting using anti-LC3 and anti-GAPDH antibodies. Representative images of LC3-II are shown (left panels).Graph (right panel) reports means ± s.e.m. of LC3-II levels after GAPDH normalization from three independent experiments; *p < 0.05, ***p < 0.001. LC3-II levels in control cells were set as 100. **(C)** LC3 level was detected by western blotting in astrocytes treated with NED-19 (1 μM) for 1, 2, and 4 h in the presence or absence of the lysosomal inhibitors E64d/pepstatin A (10 μg/mL, added 1 h before lysis). Samples were subjected to western blotting using anti-LC3 and anti-GAPDH antibodies. Representative images of LC3-II are shown (left panels). Graph (right panel) reports means ± s.e.m. of LC3-II levels after GAPDH normalization from three independent experiments. (One way ANOVA, followed by Tukey post-test).

To elucidate if NAADP is involved in the induction of autophagy by glutamate, we first confirmed that glutamate induces a release of Ca^2+^ in a NAADP-dependent manner in astrocytes. To this aim, Fluo-4-loaded cells were treated with glutamate in the presence or absence of the TPC antagonist NED-19. As shown in Figure 2A, Ca^2+^ mobilization by glutamate is dependent on NAADP-regulated channels.

We then evaluated the effect of NED-19 on glutamate induced autophagy. As shown in Figure 2B, pre-treatment treatment of cells with NED-19 did not result in an increase in LC3-II basal levels, and no further increase was observed in the presence of glutamate, indicating that autophagy induction by glutamate is inhibited in the presence of NED-19. Inhibition of basal autophagy flux by NED-19, independently of glutamate stimulation, was also confirmed by treating cells with NED-19 in the presence or absence of lysosome inhibitors (Figure 2C).

Taken together, these results indicate that glutamate activate autophagy via NAADP.

### Glutamate induces autophagy through TPCs

**Figure 3:**
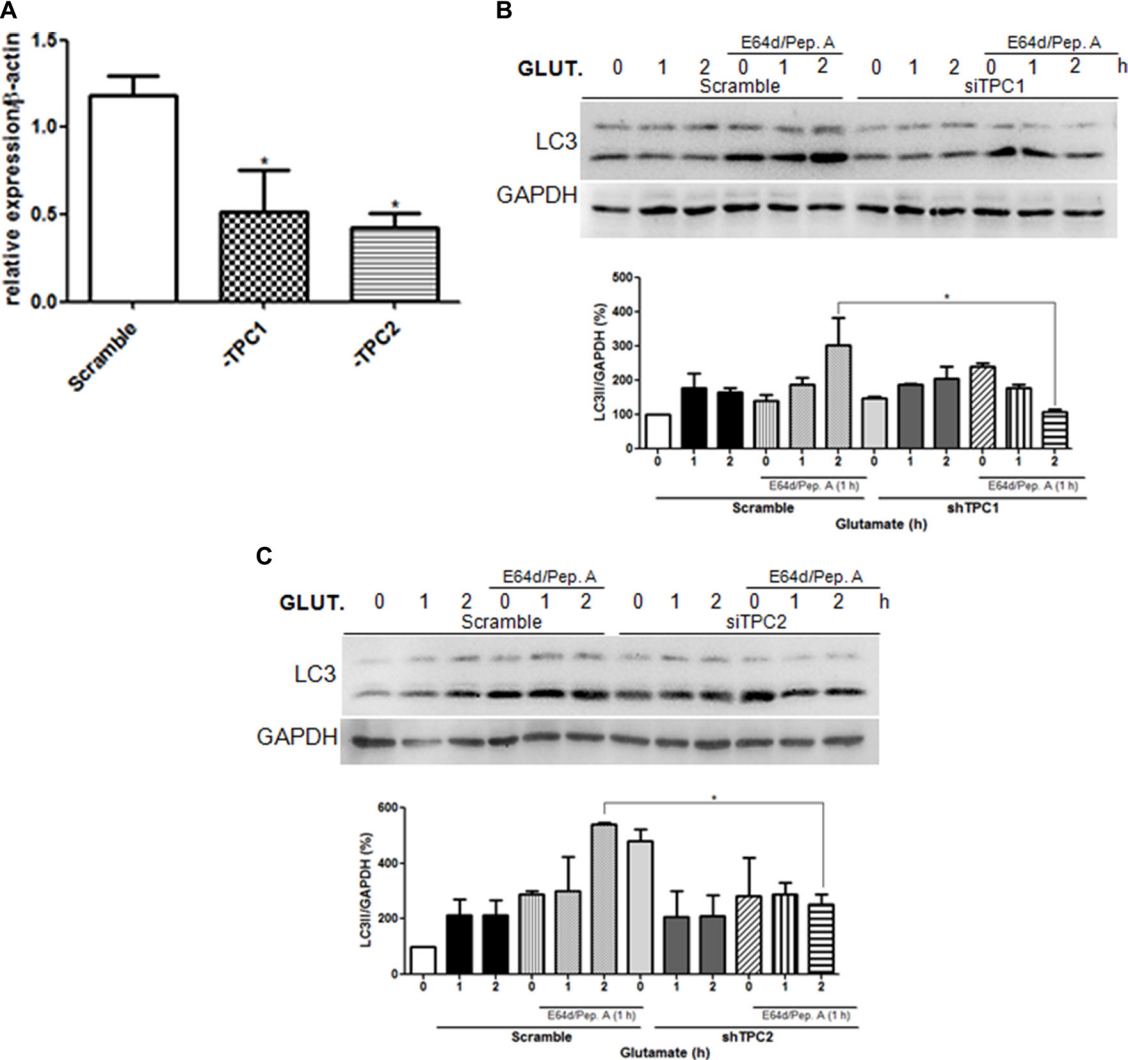
Glutamate induces autophagy through TPC1 and 2 activation The autophagic flux was evaluated in astrocytes upon downregulation of TPC1 or TPC2 using the specific siRNA oligonucleotide (siTPC1, siTPC2) in relation to control cells transfected with scramble oligonucleotide. **(A)** Analysis of TPC1 and TPC2 levels upon transfection of specific siRNA by real-time PCR. (B–C) LC3 level in astrocytes was detected by western blotting in TPC1 **(B)** or TPC2 **(C)** silenced cells treated with glutamate (10 μM) for 1, 2, 4 h in the presence or absence of the lysosomal inhibitors E64d/pepstatin A (10 μg/mL, added 1 h before lysis). Samples were subjected to western blotting using anti-LC3 and anti-GAPDH antibodies. Representative images of LC3-II are shown (upper panels). Graph (lower panel) reports means ± s.e.m. of LC3-II levels after GAPDH normalization from three independent experiments; *p < 0.05. LC3-II levels in control cells were set as 100. (One way ANOVA, followed by Tukey post-test).

Emerging studies implicate TPCs as candidate targets for NAADP within the endolysosomal system [8, 11, 21, 22, 24, 29]. To elucidate the role of TPCs in the induction of autophagy by glutamate, TPC1 or TPC2 was silenced in astrocytes (Figure 3A–3C) and in SHSY5Y cells (Supplementary Figure 1A, 1B). For the both, TPCs silencing were confirmed by quantitative PCR selective knockdown of TPC1 or TPC2 transcripts in siRNA-cells. These cells were then treated with glutamate (10 μM) for 1 or 2 h in the presence or absence of E64d/pepstatin A. Notably, TPC downregulation prevented the increase of autophagic flux induced by glutamate both in astrocytes and SHSY5Y cells, as demonstrated by the reduced increase of LC3-II levels after 2 h of glutamate in the scramble and silenced cells treated with lysosomal inhibitors. According to previous reports, we also observed that TPC2 silencing results in higher basal autophagy flux, suggesting that in absence of glutamate TPC2 might inhibit autophagy. Taken together, these results indicate that glutamate induces autophagy through activation of the NAADP-sensitive Ca^2+^ channels TPC1 and TPC2.

### Glutamate activates the upstream autophagy regulator AMPK

**Figure 4:**
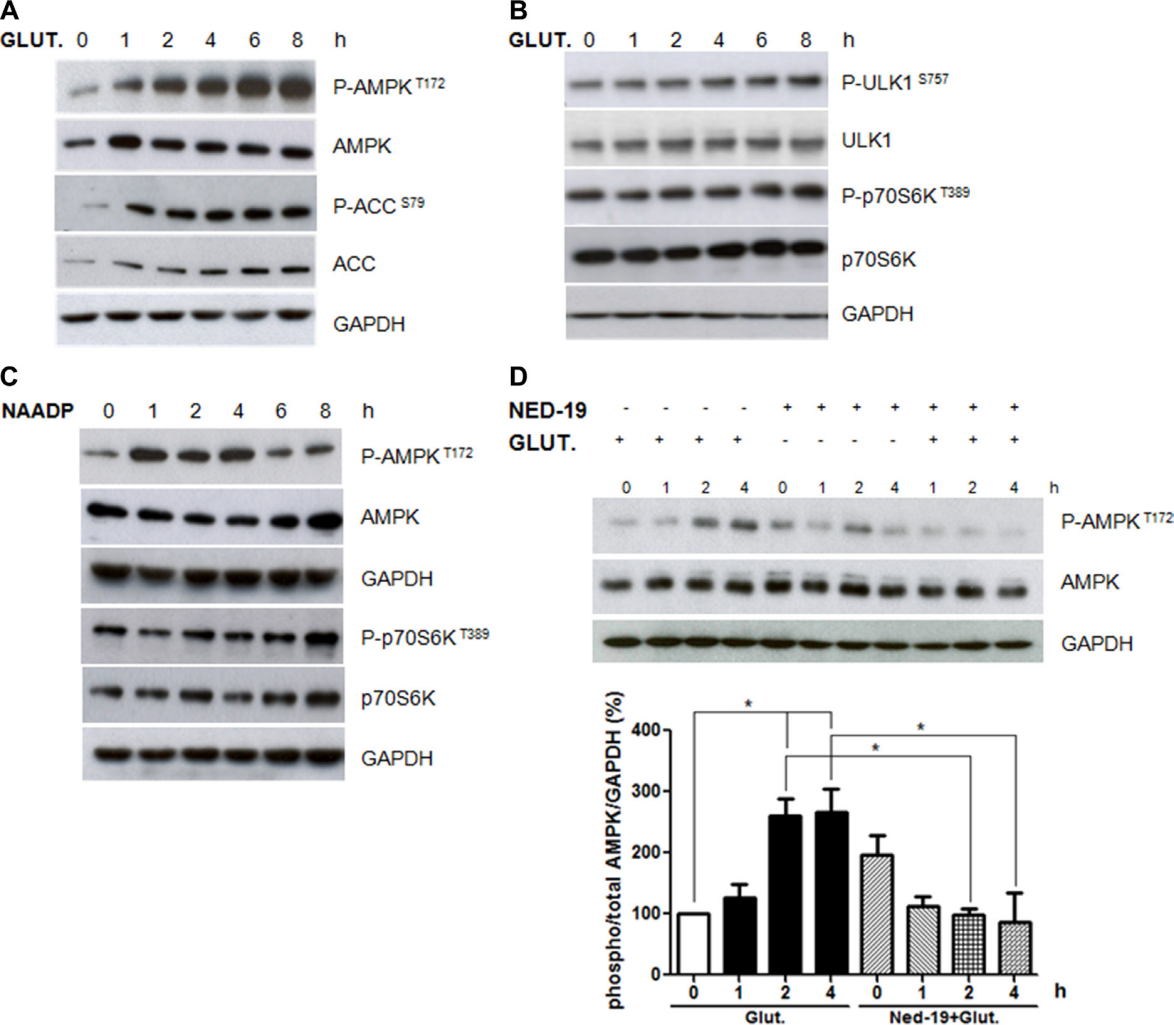
Role of AMPK and mTORC1 pathways in autophagy induction by glutamate Astrocytes were treated with glutamate (10 μM) for 1, 2, 4, 6, and 8 h. Protein extracts were subjected to western blotting analyses using antibodies for phospho-Thr172 AMPK, total AMPK, phospho-Ser79 ACC, total ACC (panel **A**), phospho-S757 ULK1, total ULK1, phospho-Thr389 p70S6K, total p70S6K (panel **B**). The expression was normalised using anti-GAPDH antibodies. **(C)** Astrocytes were treated with NAADP (100 nM) for 1, 2, 4, 6, and 8 h. Protein extracts were subjected to western blotting analyses using antibodies for phospho-Thr172 AMPK, total AMPK, phospho-Thr389 p70S6K, total p70S6K. **(D)** Astrocytes were treated with glutamate (10 μM) and NED-19 (1 μM), singly or in combination, for 1, 2, 4 h. Protein extracts were subjected to western blotting analyses using phospho-Thr172 AMPK, total AMPK and LC3. GAPDH was also analysed as a loading control. Representative images of phospho-Thr172 AMPK and total AMPK are shown (upper panels). Graph (lower panel) reports means ± s.e.m. of phospho-Thr172 AMPK levels after total AMPK and GAPDH normalization from three independent experiments; *p < 0.05. Phospho-Thr172 AMPK levels in control cells were set as 100. (One way ANOVA, followed by Tukey post-test).

Since AMP-activated protein kinase (AMPK) is known to play an important role in the regulation of Ca^2+^ stimulated autophagy [30], we evaluated the effects of NAADP-mediated glutamate signaling on AMPK activity. As shown in Figure 4A, glutamate increased the phosphorylation of both AMPK and its target ACC.

We also monitored if glutamate affects the mTOR pathway, another important upstream regulator of autophagy, by measuring the phosphorylation levels of its targets p70S6 kinase and ULK1. As shown in Figure 4B, glutamate did not inhibit p70S6 kinase and ULK1 serine 757 phosphorylation, suggesting that it activates autophagy in an mTOR-independent manner.

In agreement with a role of NAADP in autophagy regulation by glutamate, we observed that NAADP treatment induces AMPK phosphorylation, while no effect was observed on the mTOR target p70S6K (Figure 4C).

We then asked if AMPK activation by glutamate is mediated by NAADP. To this aim, we analysed the effect of glutamate on AMPK phosphorylation levels in cells pre-treated with NED-19. Importantly, NED-19 attenuated the changes in AMPK activity mediated by glutamate (Figure 4D), confirming the important role of NAADP in glutamate-regulated signalling.

## DISCUSSION

Glutamate is an excitatory neurotransmitter of the central nervous system with a major role in the complex communication network between neurons, astrocytes, oligodendrocytes, and microglia. For example, astrocytes release glutamate to regulate synaptic functions in the CNS [31–35].

Since glutamate acts by modulating a plethora of signaling pathways, the alteration of which affects many physiological brain functions. The objective of this study was to determine the mechanism by which glutamate modulates autophagy, a major determinant of protein turnover and energy source in neural cells [36]. Previous data showed that in astrocytes, glutamate increases the endogenous levels of NAADP, which evokes the release of Ca^2+^ from acidic stores [27]. Moreover, when externally applied, cell-permeable NAADP mobilizes Ca^2+^ from endolysosomes and regulates autophagy by activating TPCs [11]. As recently revised [15], several studies have been related the TPCs functionality to different roles in disease, highlighting the regulation of endolysosomal membrane traffic by local Ca^2+^ flux. The present study now provides compelling evidence that the excitatory neurotransmitter glutamate induces autophagy via NAADP and the release of Ca^2+^ from lysosomes either in astrocytes or in neuroblastoma cells.

The results show that glutamate used at a low physiological concentration (10 μM) evokes the autophagic process in astrocytes and SHSY5Y cells. Because this concentration of glutamate does not induce toxicity and cell death, it is conceivable that the induced autophagy is related to the physiological function of this neurotransmitter [37].

Autophagy is important for neuronal and glial homeostasis and its deficiency in dopaminergic neurons results in increased size of axon profiles, which alters the presynaptic structure and neurotransmission, for example [36]. This indicates that physiological and stimulatory conditions lead to autophagy induction, which may act selectively to remove damaged organelles in a cytoprotective process [36, 37]. Glutamate is also a metabolic substrate, and particularly in astrocytes it exerts a critical role in energy production, by recycling the excessive glutamate released in synaptic cleft and converting to α-ketoglutarate and ammonia [38] therefore, in both neurons and glia, it is a source of energy, which is important for the storage of neurotransmitters as well as brain function [35]. In other systems such as liver and pancreas, the gain-of-function mutation in glutamate dehydrogenase (GDH) cause the hyperinsulinism/hyperammonemia (HI/HA) syndrome [39]. It was demonstrated that hyperammonemia can increase the autophagy markers such as beclin-1, LC3II and p62 and contributes to muscle loss in sarcopenia with cirrhosis condition [40]. By contrast, a high concentration of glutamate leads progressively to cell death by apoptosis or other death mechanisms [41, 42]. Indeed, overstimulation of N-methyl-D-aspartate (NMDA) receptors by glutamate can produce massive Ca^2+^ entry, which is taken up by mitochondria and leads to excitotoxicity [43]. NMDA overactivation may contribute to altered Ca^2+^ homeostasis in neurodegenerative diseases [42, 44, 45]. The data reported in this study indicate that a low concentration of glutamate stimulates autophagy and acts as a pro-survival mechanism under physiological conditions. In fact, we demonstrated that TPC1 or TPC2 downregulation impairs the increase of lipidated LC3 upon glutamate treatment. This indicates that both TPC1 and TPC2 activation are important for glutamate signalling and stimulation of autophagosome formation. These data are consistent with previous reports showing that inhibition of the TPC2/NAADP/Ca^2+^ pathway impairs basal autophagy in HeLa cells and TPCN2^−/−^ mice [26].

In this study, we also investigated the mechanism by which Ca^2+^ release from lysosomes induced by NAADP modulates autophagy flux. Our data indicate that the AMPK/ACC pathway is stimulated by both glutamate and NAADP, whereas NED-19 abrogates this activation.

AMPK is a crucial modulator of the autophagic process in response to energy deprivation and changes in Ca^2+^ levels. AMPK can induce autophagy by acting via two different pathways, directly, by phosphorylating the proautophagic factor ULK1 [46, 47], and indirectly, by negatively regulating the mTOR complex targeting them TOR inhibitors tuberous sclerosis complex 1 and 2 (TSC1/2) and the mTOR activator Rheb, a Ras homologue GTP binding protein enriched in the brain [30, 43, 49]. In our system, the AMPK-dependent activation of autophagy by glutamate appears to act in a mTOR-independent manner, because the phosphorylation of its targets p70S6K1 and serine 757 ULK1 were found unchanged. This is consistent with studies performed in highly metabolic cells, such as skeletal muscle, in which activation of autophagy by AMPK occurs without interfering with the pleiotropic function of mTOR required to sustain physiological activities. It was reported that AMPK activation affects glutamate metabolism in tricarboxylic acid (TCA) cycle in astrocytes [50], which may have implications in energy availability in CNS. Together, these results suggest that AMPK is an important sensor for maintaining glutamatergic neurotransmission by modulating autophagy.

In conclusion, the present findings support a novel physiological role for glutamate, which, via NAADP-mediated TPCs activation, leads to the release of lysosomal Ca^2+^, and AMPK-dependent autophagy induction. In light of these results, we propose that the glutamate/NAADP-induced autophagy signalling is a promising pathway for targeting in metabolic processes in the SNC.

## MATERIALS AND METHODS

### Drugs

NAADP-AM and NED-19 were synthesised as previously described [51, 52]. Glutamate, E64d, pepstatin A and G418 were purchased from Sigma-Aldrich Chemical Co., St Louis, MO, USA.

### Cell culture

Astrocytes were immortalized as described previously [53]; briefly, the primary astrocytes from rat cortex were transfected with 3 μg of pSV3-*neo*, a plasmid containing the SV40 T antigen and the neomycin resistance gene. SHSY5Y cells were cultured in DMEM (Sigma-Aldrich) supplemented with 10% FBS (Life Technologies), 2 mM L-glutamine, and 1% penicillin/streptomycin solution at 37°C under 5% CO_2_. Lysosome activity was inhibited with E64d and pepstatin A (10 μg/mL). The plasmids *short hairpin* pSUPER + GFP vector, GFP-*Scramble* GFP-shTPC1 or GFP-shTPC2 were obtained as previously cloned (Oligoengine) [21]. SHSY5Y cells were transfected with each plasmid (2 μg/mL) using Lipofectamine LTX (Life Technologies) and according to the manufacturer's instructions. Transfected cells were then selected with 0.4 mg/mL G418 for 1 month.

### Plasmids and retroviral infection

The myc-tagged, full-length wild-type TPC1 and TPC2 constructs were kindly provided by Sandip Patel (UCL, London, UK) and cloned into a modified version of the pLPCX vector (Clontech) [54]. The LC3 construct mCherry-LC3B was previously described [55]. For virus production, 15 μg retroviral vector as co-transfected with 5 μg expression plasmid for the vesicular stomatitis virus G protein into the 293 gp/bsr cell line using the calcium phosphate method. After 48 h, the supernatant containing the retroviral particles was recovered and supplemented with 4 μg/mL polybrene and stored at –80°C.

### Antibodies

The primary antibodies used in this study were as follows: the rabbit antibodies anti-LC3B, phospho-Ser79 ACC, total ACC, phospho-Thr172 AMPK, total AMPK, phospho-Thr389 p70S6K, total p70S6K and phospho-serin757 ULK1 (Cell Signaling); anti-ULK1 (Santa Cruz Biotechnology); anti-TPC1 and TPC2 (Abcam); anti-GAPDH (Calbiochem); anti-β-actin (Sigma-Aldrich).

### RNA interference

RNA interference in astrocytes was performed using the following RNA oligonucleotide duplexes from Life Technologies: TPCN1 and TPCN2. A total of 1.0 × 10^5^ cells/well were transfected with 20 nmol siRNA oligonucleotides in 6-well plates using Lipofectamine RNAiMAX (Life Technologies) following the supplier's instructions. For rat astrocytes, the sequences for each transcript were TPCN1 forward (5′-GGAUCCUAGUGGA GACAUUTT-3′) and reverse (5′-AAUGUCUCCACUAG GAUCCAG-3′); TPCN2 forward (5′-GGAAACCUCUU GUAUUUTT-3′) and reverse (5′-AAAUAGACAAGAG GUUUCCCA-3′); Gene silencing was verified by real-time PCR analysis 48 h after transfection.

### Confocal microscopy

Autophagic flux was measured in the mCherry-LC3B stable cell line. Astrocytes expressing Cherry-LC3 were grown on a coverslip, treated with different conditions and fixed with 4% paraformaldehyde for 15 min in PBS. The coverslips were mounted with antifade (SlowFade; Life Technologies) and examined under a confocal microscope (TCS SP2; Leica). The results report the number of LC3 puncta per cell. For quantification of red (Cherry) mCherry-LC3 (td-tag-LC3) puncta, pictures were captured at 60× magnification on a confocal microscope, and the number of red puncta analysed as described above from (30–40) randomly selected cells per experiment and condition.

### Western blotting assays

Cells for each different experimental protocol were lysed in RIPA (150 mM NaCl, 1% NP-40, 0.5% deoxycholic acid, 0.1% SDS, 50 mM Tris pH 8.0, and 2 mM MgCl_2_). Protease and phosphatase inhibitors (protease inhibitor cocktail plus 5 mM sodium fluoride, 0.5 mM sodium orthovanadate, 1 mM sodium molybdate, 50 mM 2-chloroacetamide, 2 mM 1,10-phenanthroline monohydrate, and 0,5 mM PMSF; Sigma-Aldrich) were added. Lysates were incubated at 4°C for 30 min. After centrifugation at 4°C for 10 min at 13,000 rpm to remove insoluble debris, the protein concentrations were determined using a Bradford assay with bovine serum albumin as the reference standard (Biorad). Equal amounts of protein (10 μg) were re-suspended in SDS-PAGE sample buffer. The samples were then separated on NuPAGE Bis-Tris gel (Life Technologies) and electroblotted onto nitrocellulose (Protran, Schleicher & Schuell) or PVDF (Millipore) membranes. Blots were incubated with primary antibody in 5% non-fat dry milk in PBS plus 0.1% Tween-20 overnight at 4°C. Detection was achieved using a horseradish peroxidase-conjugate secondary antibody (Jackson Immuno Research Laboratories) and visualised with ECL (GE Healthcare).

### Ca^2+^ measurements

Astrocytes were plated on coverslips and incubated with 3 μM Fluo-4AM (Molecular Probes, Eugene, OR, USA) for 30 minutes in a microscopy buffer containing (in mM): 130 NaCl, 5.36 KCl, 0.8 MgSO_4_, 1 Na_2_HPO_4_, 25 glucose, 20 HEPES, pH 7.3. Fluo-4 AM fluorescence was acquired at 3 s intervals with alternate excitation at 494 nm and emission at 506 nm. Using an inverted fluorescence microscope Nikon TE 300; Nikon, Osaka, Japan) coupled to a CCD camera (CoolSnap, Princeton Instruments, Princeton, NJ). Images were analyzed with BioIP software (Anderson Eng, Delaware, USA).

### Real Time PCR

RNA was extracted by using Trizol reagent (Invitrogen). cDNA synthesis was generated using a reverse transcription kit (Promega) according to the manufacturer's recommendations. Quantitative PCR reactions were performed with the Rotor-Gene 6000 (Corbett Research Ltd) thermocycler. The Maxima SYBR Green/ROX qPCR Master Mix (Thermo Scientific) was used to produce fluorescently labeled PCR products. Primer sets for all amplicons were designed using the Primer-Express 1.0 software system (Roche): For SHSY5Y were used: human TPC1 forward (5′-TCCGGATGGAA CTTGTTTG-3′) and reverse (5′-GCAGGACCACGA TGAAATAG-3′); human TPC2 forward (5′-TGGTGGAC TGTCGGTATT-3′) and reverse (5′-AAACCGAGGATG GCAAAG-3′); human GAPDH forward (5′-CGCTTCGCT CTCTGCTCCT-3′) and reverse (5′-CCGTTGACTCCG ACCTTCAC-3′). The oligonucleotide primer sequences to TPCs from rat astrocytes were described previously [21].

### Statistical analysis

All values are represented as the mean ± s.e.m. Significance was tested by ANOVA followed by Dunnett test for comparisons with the experiment control. Multiple comparisons among group mean differences were assessed with Tukey post-test. Differences were considered significant when **p* <* 0.05.

## SUPPLEMENTARY MATERIALS


