# Supplementary material for: Glutamate induces autophagy via the two-pore channels in neural cells
Source: Oncotarget. 2016 Dec 31;8(8):12730–40. doi: 10.18632/oncotarget.14404 (PMC5355049; doi:10.18632/oncotarget.14404)
Supplement: Supplementary file 1 [file oncotarget-08-12730-s001.pdf]

Glutamate induces autophagy via the two-pore channels in neural cells

Supplementary Materials

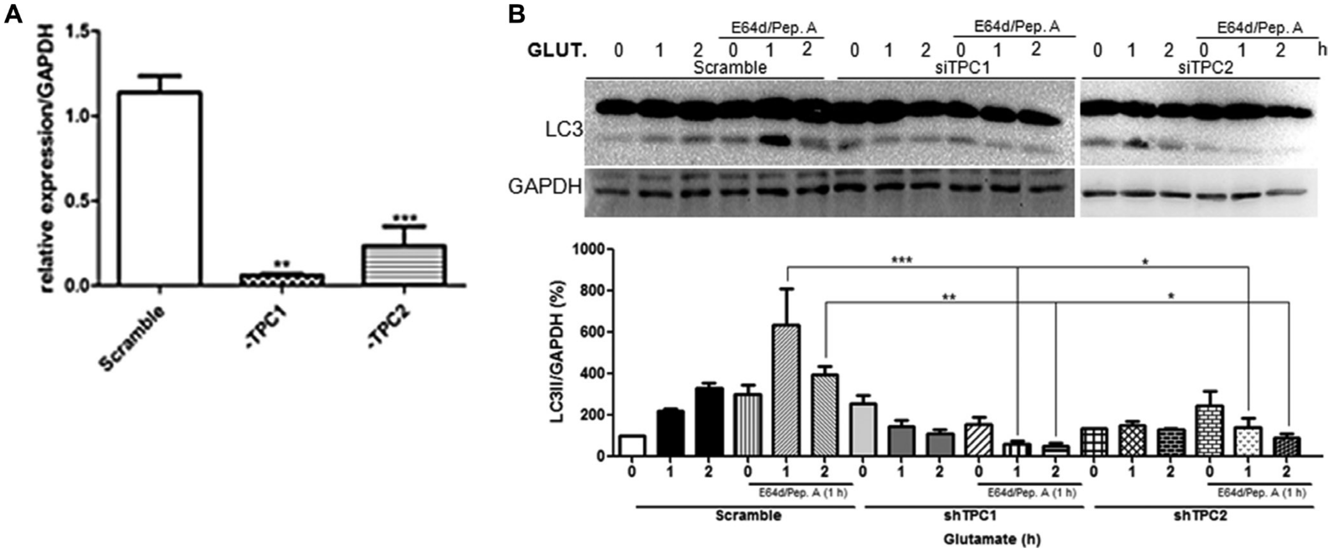

Supplementary Figure 1: Glutamate induces autophagy through TPC1 and 2 activation in SHSY5Y cells. The autophagic flux was evaluated in SHSY5Y cells upon downregulation of TPC1 or TPC2 using the specific siRNA oligonucleotide (siTPC1, siTPC2) in relation to control cells transfected with scramble oligonucleotide. (**A**) Analysis of TPC1 and TPC2 levels upon transfection of specific siRNA by real-time PCR. (**B**) LC3 level in SHSY5Y was detected by western blotting in TPC1 or TPC2 silenced cells treated with glutamate (10 µM) for 1, 2, 4 h in the presence or absence of the lysosomal inhibitors E64d/pepstatin A (10 µg/mL, added 1 h before lysis). Samples were subjected to western blotting using anti-LC3 and anti-GAPDH antibodies. Representative images of LC3-II are shown (upper panels). Graph (lower panel) reports means ± s.e.m. of LC3-II levels after GAPDH normalization from three independent experiments; $*p < 0.05$, $**p < 0.01$, $***p < 0.001$. LC3-II levels in control cells were set as 100. (one way ANOVA, followed by Tukey post-test).
